# Chicken’s best friend? Livestock guardian dog bonding with free-ranging chickens

**DOI:** 10.1093/tas/txad014

**Published:** 2023-02-07

**Authors:** R A McKellar, T L Kreplins, P A Fleming

**Affiliations:** Terrestrial Ecosystem Science and Sustainability, Harry Butler Institute, Murdoch University, Murdoch, Western Australia, Australia; Terrestrial Ecosystem Science and Sustainability, Harry Butler Institute, Murdoch University, Murdoch, Western Australia, Australia; Department of Primary Industries and Regional Development, Northam, Western Australia, Australia; Terrestrial Ecosystem Science and Sustainability, Harry Butler Institute, Murdoch University, Murdoch, Western Australia, Australia

**Keywords:** alpaca, behavior, Maremma, predator, predation, poultry

## Abstract

Growth in the free-range and pastured egg industries has increased globally, necessitating improvements in predator control. Some egg producers are turning to the use of livestock guardian dogs (LGD; *Canis familiaris*) to protect hens from predation. We worked on a property where pastured layer hens were protected by two Maremma LGDs that were released from their chicken enclosure for 2–3 nights a week. GPS tracking showed that the dogs were more strongly bonded to people than the chickens, spending most of their time at night (96.1% of location data) close to the farmhouse and only 0.09% near their chicken paddock. Despite this lack of attendance, we found no change in the paddock space use by chickens with or without the dogs present (*P* = 0.999). Furthermore, camera trapping revealed 40 red fox (*Vulpes vulpes*) events over the 46-d monitoring period, with less fox activity on nights when the LGDs were allowed to roam the property and motion-activated spotlights were also deployed (*P* = 0.048). An online survey of 59 poultry producers found strong belief in the effectiveness of LGDs, although half the respondents (52%) indicated that they were still experiencing predation issues. There was no association with the reported degree of human bonding of their LGDs, but respondents were more likely to report current issues with predators if they owned 100 or more chickens (*P* = 0.031). The present case study as well as the farmer survey have identified that LGDs can be strongly bonded to people. Although there was no evidence of subsequently increased risk of predation, bonding with people could draw LGDs away from the animals they should be defending, with predation risk for poultry likely to depend on how far away LGDs move from their livestock.

## Teaser

Guardian dogs can protect chickens from predation, whether they are kept in with the chickens or allowed to patrol around the property at large.

## INTRODUCTION

Growing consumer demand for high quality and high animal welfare products is driving the move away from intensive cage egg production toward outdoor production systems. For example, in the United Kingdom, cage eggs decreased from 43% to 29% of total production over 2.5 yr (gov.uk, 2022) and there has similarly been a move away from cage eggs in the United States (USDA, 2022). In Australia, more than half (58%) of eggs sold through supermarkets are from outdoor systems (Australian Eggs, 2022), and while the number of cage eggs sold over the last financial year decreased by 17%, specialty eggs—including organic certified and pastured eggs—increased by 11% (Australian Eggs, 2022; see https://www.australianeggs.org.au/farming for definitions). Over this same time, Australian consumers paid twice the price for specialty eggs than cage eggs (Australian Eggs, 2022). This specialty market can therefore represent good value for the producers, who then invest in methods to maximize welfare for layer hens.

Increasing numbers of layer chickens kept outdoors has meant a larger number of hens can be vulnerable to predators (e.g., Moberly et al., 2004; Knierim, 2006; Bestman and Bikker-Ouwejan, 2020), with predation being the primary threat recorded across 41 Australian free-range flocks (Singh et al., 2017). Electrified fencing is effective in keeping chickens in as well as predators out, while netting roofing offers additional protection against raptors, which can cause substantial losses (Bestman and Bikker-Ouwejan, 2020). Other methods, such as chemical, auditory, or visual deterrents could have some potential (Van Eeden et al., 2018b; Smith et al., 2021), although predators typically become accustomed to deterrents over time (Khorozyan and Waltert, 2019) or can even be attracted to their presence (Hall and Fleming, 2021). For example, a range of different lighting options have been trialed, with varying efficacy as a predator deterrent (e.g., Lesilau et al., 2018; Adams et al., 2020; Hall and Fleming, 2021). Although predators are likely to quickly become habituated to passive deterrents (Khorozyan and Waltert, 2019), active defenses such as motion-activated devices or reinforcement of the deterrent cue with other modes (e.g., combining visual with auditory cues) (Biedenweg et al., 2011) could improve their effectiveness. There is not much empirical data on the effectiveness of predator deterrents, with few published studies testing combinations of methods (Eklund et al., 2017; Khorozyan and Waltert, 2019) or applying these to the protection of poultry (Smith et al., 2000; Beranger et al., 2007).

Across the globe, many livestock producers have turned to the use of livestock guardian dogs (LGDs; *Canis familiaris*) for predator control (McGrew and Blakesley, 1982; Andelt, 1999; van Bommel and Johnson, 2012). LGDs can be used in conjunction with conventional methods of livestock management, and in a variety of agricultural contexts (Andelt, 1999). Their use particularly appeals to producers who are searching for a cost-effective, non-lethal form of predator control that can give them confidence that their livestock is being protected at all times (Gehring et al., 2010; van Bommel, 2010; van Bommel and Johnson, 2012, 2015). Despite growing interest in the use of LGDs for predator control, there are still questions around potential aversive impacts of LGDs (e.g., Drouilly et al., 2020; Smith et al., 2020; Marker et al., 2021), the best management of LGDs for most effective reductions in predation (van Bommel, 2010), or even how they guard livestock (van Bommel and Johnson, 2017). Most published studies of LGDs to date have been carried out around the protection of sheep (*Ovis aries*) (Van Eeden et al., 2018b). When working with sheep and having freedom of movement, LGDs spend the majority of their time with their livestock, leaving only for short periods (van Bommel and Johnson, 2014, 2015). It appears to be the direct presence of LGD that deters predators (McGrew and Blakesley, 1982; Allen et al., 2017) rather than the establishment of territorial boundaries from which predators are excluded (but see van Bommel and Johnson, 2015). For example, half of interactions between LGD and wolves occur at a distance greater than 300 m from the sheep flock (Landry et al., 2020), indicating that the dogs move beyond their flocks to respond to the presence of potential threats. LGD may therefore still be able to provide protection even at a distance from their livestock; their perceived absence could, however, influence the behavior of the livestock they are tasked to protect.

There have been few studies of the efficacy of guardian animals in predator deterrence for poultry, including a case study (van Bommel, 2013), two surveys of farmers using LGDs to protect poultry (Otstavel et al., 2009; van Bommel and Johnson, 2012), and a manipulative study comparing the influence of fox cues on space used by the dogs (Roddick et al., 2022). Published findings from guarding sheep cannot be generalized to how LGDs are likely to protect poultry flocks. In addition to marked differences in the numbers of animals for these production systems and the spatial scale over which the livestock are distributed, the body size and behavior of sheep are very different from that of poultry. Furthermore, egg production requires substantial continuous human interaction to manage the layer hens, and the degree of bonding can shift away from the chickens toward humans (van Bommel, 2010). Studies on LGDs have focused on their ability to reduce predation (Coppinger et al., 1988; Andelt and Hopper, 2000; van Bommel and Johnson, 2012) with little attention given to LGD bonding with livestock vs. humans and how this might influence their efficiency. For all these reasons, increasing understanding of LGD behavior around free-range poultry is therefore warranted.

In this study, we worked with a commercial egg producer who ran pastured chickens. The producer had experienced hen losses to red foxes (*Vulpes vulpes*) prior to obtaining two Maremmas and two alpacas (*Vicugna pacos*), which each managed one of three chicken paddocks; the third chicken paddock had no guardian animals. As part of their normal husbandry practice, for 2–3 d each week, the LGDs were let out of the chicken paddock and allowed to free-roam the whole property for their welfare and also to provide protection across the whole property. This situation enabled us to carry out a pilot study examining the following experimental questions:

1. Are livestock guardian dogs (LGDs) bonded to their chickens, even when they are free to roam? We compared the LGD’s movements and space use when they were free to roam across the property to test whether they remained close to their chickens.
2. Does the presence of LGDs increase paddock use by chickens? We compared the distribution of chickens in response to the presence or absence of the LGD in their paddock.
3. Is fox activity influenced by the presence of free-roaming LGDs and/or motion-activated sensor spotlights? We examined whether the activity of LGD could be reinforced with the addition of motion-activated visual (lighting) deterrents.
4. What factors influence the effectiveness of LGDs? We supported our on-farm data with an online survey of poultry farmers to identify factors that influenced LGD effectiveness (i.e., reduction in poultry predation).

## METHODS

This project was approved by the Murdoch University Animal Ethics Committee (R3173/19) and Human Ethics Committee (2019/121).

### Study Site

Fieldwork was conducted from 8 July to 23 August 2019 at Blue Tractor Farm in Glen Mervyn, Western Australia. The farm had been working as a commercial egg farm for about 2 yr at the time of this study, keeping pastured Isa Brown chicken (*Gallus gallus domesticus*) layers. The owner had experienced substantial losses to predators in the year before acquiring the LGDs: on one night, 35 chickens were killed by foxes, another 15 on a separate occasion, and foxes were regularly seen both day and night. In addition, an estimated ~15 chickens were seen killed by raptors. Furthermore, uncounted numbers of chickens that flew out of the fenced paddocks (and could not be re-captured) were also likely predated, as they did not remain present for many days. In the subsequent year of operation, after getting two alpacas and then two LGDs, there were no reported losses to either foxes or raptors, and fewer foxes had been noticed on the property (a distinction in timing between the alpacas and LGDs was not made).

**Figure 1.**
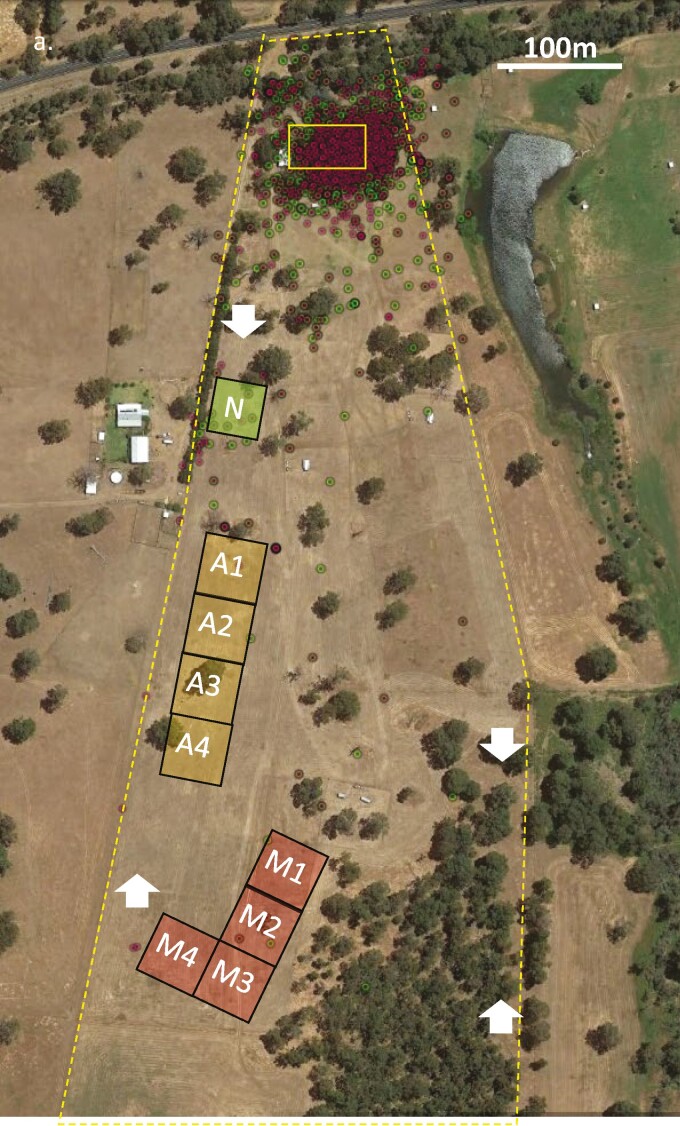
Study site displaying property boundaries (dashed line), farmhouse (rectangle at top of image), and approximate location and size (50 × 50 m) at the time of the present study of the chicken paddocks (squares) that were guarded by two Maremma LGDs (M1–M4) or two alpacas (A1–A4), with numbers (1–4) denoting the weekly paddock movement order, or no livestock guardian animal (N; did not move during the study period). Camera traps were placed at the corners of each chicken paddock facing down the fences (total = 12), and an additional four camera traps were paired with four motion-activated spotlights around the perimeter of the property (white arrows indicate the location and direction of the spotlights). Red and green dots show GPS data points for movements of the two LGDs when released from their chicken paddock (M1–M4) over the 6-wk data gathering period.

**Figure 2.**
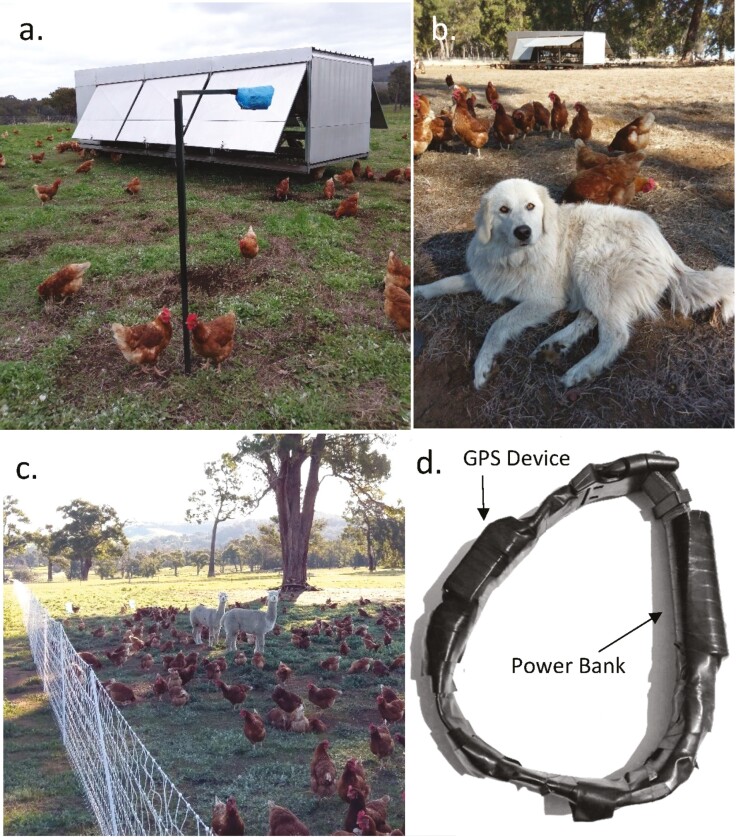
(a) Paddock showing a chicken caravan (for egg laying and night-time roosting) in the background and the downward-facing camera traps (with protective waterproof covering—disposable plastic blue shoe cover) used to record chicken activity in the foreground. (b) Maremma LGD in front of chickens. (c) Two guardian alpacas, showing the portable electrified fencing and open paddock layout of the property. (d) GPS tracking collars are used to monitor LGD movements.

The property had three separate 50 × 50 m chicken paddocks (Figure 1) surrounded by solar-powered electric poultry fences and each containing a “chicken caravan” for egg laying and night-time roosting (Figure 2a). Paddock “M,” the focus of our work, contained two Maremma LGDs and 450 ~9-month-old chickens (Figure 2b). The 2-yr-old sibling LGDs (male: Hiro and female: Coco) had been acquired 1 yr previously from another open pasture chicken farm, where they had been raised amongst chickens. Paddock “A” contained two alpacas (which always remained within the confines of their paddock) and 500 ~6-month-old chickens (Figure 2c). There has been some reported success in protecting flocks of sheep by llamas (*Lama glama*) (Meadows and Knowlton, 2000) and the smaller alpacas (Mahoney and Charry, 2007); however, we are not aware of any test of the guardian value for poultry and we also did not have sufficient data to carry out a robust experimental design on this property. Both Paddock M and Paddock A were moved every 2 to 3 wk to an adjacent 50 × 50 m area for fresh green pick and foraging opportunities. Paddock “N” contained no guardian animal and the oldest chickens at ~18-months-old. Paddock N was not moved during the study as the chickens were being sold off; there were 68 chickens for the first 12 d of monitoring, 47 for the next day and then 25 chickens for the last 2 d of monitoring for this paddock.

### Are LGDs Bonded to Their Chickens Even When They are Free to Roam?

To track the movements of the LGDs when they were allowed out of their chicken paddock to roam the rest of the farm, i-gotu GT-120 (MobileAction, Mobile Action Technology Inc, Taipei, Taiwan) GPS tracking devices were configured to record a GPS location reading every 3 min, or every 10 s if the movement of the device increased to 10 km/h. Each was powered by a 2600 mAh portable power bank (Digitech, Epsilon Digitech LLP, Gujarat, India) which kept the GPS device operational for approximately 5–8 d, depending on reading frequency and weather conditions. The GPS device and power bank was attached to a large domestic dog collar using weather-resistant tape (Gaffa Tape, Scotch Tape) (Figure 2d). Four collars were constructed; each LGD wore a collar and collars were swapped each week to continuously monitor their activity for 7 wk.

GPS data were downloaded using the @trip software (Mobile Action Technology Inc) and then exported into Microsoft excel (Microsoft Corporation). Each data point was attributed as a day (06:30 h–18:30 h daylight hours at the time of this study) or night (18:30 h–06:30 h). We grouped times by LGD treatment (LGDs confined to their paddock “dogs in”; LGDs free to roam about the farm “dogs out” for 2–3 days and nights each week). Using Pythagorean calculation in excel, eastings and northings were used to calculate “separation distance” from their chicken paddock when the dogs were out as the distance of each data point from the centroid location of their current chicken paddock. We compared separation distance between night and day using Kruskall–Wallis tests in R (R Core Team, 2018); this statistic was used due to the skewed distribution of the data. We also compared their speed of movements and separation distance (two separate analyses) for nights with and without the motion-activated spotlights (“spotlights on”/”spotlights off”).

### Does the Presence of LGDs Increase Paddock Use by Chickens?

To determine which parts of the paddock were used by chickens and whether their activity was influenced by the presence of the LGDs, we positioned camera traps at various locations through the chicken paddocks. Eight Reconyx HC500 Hyperfire remote infrared sensor camera traps (Reconyx Inc., Holmen, WI, USA) were attached, face down, to 1.8 m-tall gantry posts (Figure 2a) at varying intervals dividing the distance between the caravan and each corner of the paddock (Appendix A). This arrangement allowed the design to be modified as the paddock shifted location and changed shape and area slightly over time. Each camera trap was set to capture an image every 5 min on a time lapse setting (i.e., 12 images per hour) and monitoring was continued for 20 d. Batteries and SD cards were checked at least once a week.

The number of chickens in each image was counted, along with an assessment of whether shade was present (>50% of the field of view was shaded). The total number of chickens seen on each camera trap was summed for each hour (“chicken activity”) and the proportion of photos showing shade present was calculated for each hour (“shade availability”). To test whether LGD presence increased the distance chickens would move away from the protective cover of the laying caravan, we carried out a generalized linear model analysis (using the glm function in the R package “lme4,” Bates et al., 2012) with chicken activity as the dependent variable, LGD treatment (“dogs in”/“dogs out”) by distance (camera trap to the caravan; m) as an interaction term, and shade availability as a covariate. The chicken activity data did not meet the assumptions of a normal distribution and therefore we applied a tweedie family link function (Dunn, 2017) to these data, which met the requirements of normally-distributed residual values (R package DHARMa; Hartig, 2020).

### Is Fox Activity Influenced by the Presence of Free-Roaming LGDs or Motion-Activated Sensor Spotlights?

To detect fox activity around the chicken paddocks, we positioned passive infrared camera traps (HC500 Hyperfire, Reconyx Inc., Holmen, WI, USA) 3 m away from the corner of each of the three chicken paddocks (total of 12 camera traps), with the camera traps facing down the sides of the paddock. Camera traps were attached to 0.9 m star pickets at a height of ~0.6 m. Each camera trap was set on “Rapidfire” to take three photos per trigger.

Four motion-activated spotlights (Dual solar LED security lights, Lectro) were positioned 50–150 m from the chicken paddocks near the property boundary fence. Sensitivity of the spotlights was adjusted to activate in low light conditions, and they were programmed to stay on for 2 min when activated. Each spotlight was paired with a camera trap placed within 1 m of it. In addition to being activated by heat in motion, the camera traps were also programmed to take an image on time lapse every 1 min, which allowed us to distinguish when the spotlights had been activated but the camera trap had not captured the trigger. While the spotlights and camera traps were in position throughout the monitoring period, the spotlights were switched on or off for alternate weeks (“spotlights on”/”spotlights off”).

Camera traps had their batteries and SD cards checked every 3–4 d and were left in position for a total of 46 d. Photos were checked for the presence of foxes, with independent fox sightings counted if photo captures on the same camera trap were separated by a minimum of 5 min. We only had one instance of two fox sightings within 5 min, but these were on different (adjacent) cameras and so were counted as independent events; all other sightings were more than 11 min apart. Pearson’s Chi-squared test (manually calculated in Excel) was used to compare the number of fox events on camera trap for the four combinations of the LGD (“dogs in”/”dogs out”) and spotlight (“spotlights on”/”spotlights off”) treatments, with expected values calculated by assuming an equal proportion of fox sightings per night monitoring for each of the four treatments. We also compared pairwise for LGD treatment nights (“dogs in” vs. “dogs out” on nights without spotlights), or spotlight treatment nights (“spotlights on” for nights with and without the dogs out) using the same statistical approach.

### What Factors Influence Effectiveness of LGDs? A Poultry Farmer Survey

We distributed a survey through SurveyMonkey for a period of 2 months (mid-September to mid-November 2018) targeting LGD and chicken farming Facebook groups (Blue Tractor Farm Pastured Eggs, LGDs Australia, West Coast Maremma Rescue, Australian Pasture Fresh, Livestock Guard Dog Rescue in Australia, and PROOF—Pasture Raised on Open Fields). We asked respondents to also share the link to the survey. The survey included separate questions for respondents who identified as 1) currently having LGDs, 2) who had used LGDs but no longer did, and 3) who had never used LGDs. Otherwise, the questions were kept as consistent as possible among the three groups to gain as many comparable views as possible. The questions asked included what predator issues the farm faced, how they dealt with those issues, what they viewed as the effectiveness and cost of using LGDs, and whether they considered them worth it. A full list of the questions can be found in the Appendix B.

When processing survey results, the single response from New Zealand was grouped together with responses from Australia, and responses from North America (USA and Canada) were grouped together. We compared poultry numbers between Australian and North American respondents using a two-sample *t* test assuming unequal variances in R.

We grouped responses to the question of whether they had persisting predator issues as “yes” or “no.” Respondents who selected the options “some,” “occasional,” or “rarely” were grouped with respondents who replied “yes” (i.e., were all categorized as still having predation issues). All these respondents had LGD. Post hoc assessment of text responses to the survey indicated that current problems experienced were directly due to livestock predation issues. We carried out a binomial glm in R with dependent variable being persisting predator issues, and predictor variables 1) the number of LGDs, 2) number of poultry (log-transformed; when ranges of bird livestock numbers were given, the higher value was used), and 3) whether the dogs worked inside a fenced area. We selected these predictor variables as having the best balance of representation in the dataset and the least missing data. We compared models with different combinations of these predictor variables using the *dredge* function in the R package “MuMIn” (Barton and Barton, 2020) which indicated that the full global model had the best weight of support (AIC value). The distribution of residuals for this model (R package DHARMa) indicated we could accept the use of a gaussian fit for this model.

Because of missing data in responses, we carried out additional separate statistical analysis for reported persisting predator issues by individual predictor variables using Pearson’s Chi-squared tests (manually calculated in Excel), separately comparing between 1) respondents who selected that their dogs were more bonded to “livestock” or “people,” 2) where the LGDs received <1 h per week of human interaction, <1 h per day, or >1 h per day, 3) by dog breed (Maremma or other), 4) whether the dogs were reported to work alone or in groups, and 5) for respondents who identified training in their advice to farmers using LGDs for the first time. In all these tests, the expected values were calculated assuming an equal proportion of responses between respondents who reported persisting predator issues or not.

Finally, we compared whether LGDs had injured or killed livestock between groups of respondents who reported their LGDs spent all of their time with the livestock or not using a Pearson’s Chi-squared test. Expected values were calculated assuming an equal proportion of livestock injuries for the two groups.

## RESULTS

### Are LGDs Bonded to Their Chickens When They are Free to Roam?

**Figure 3.**
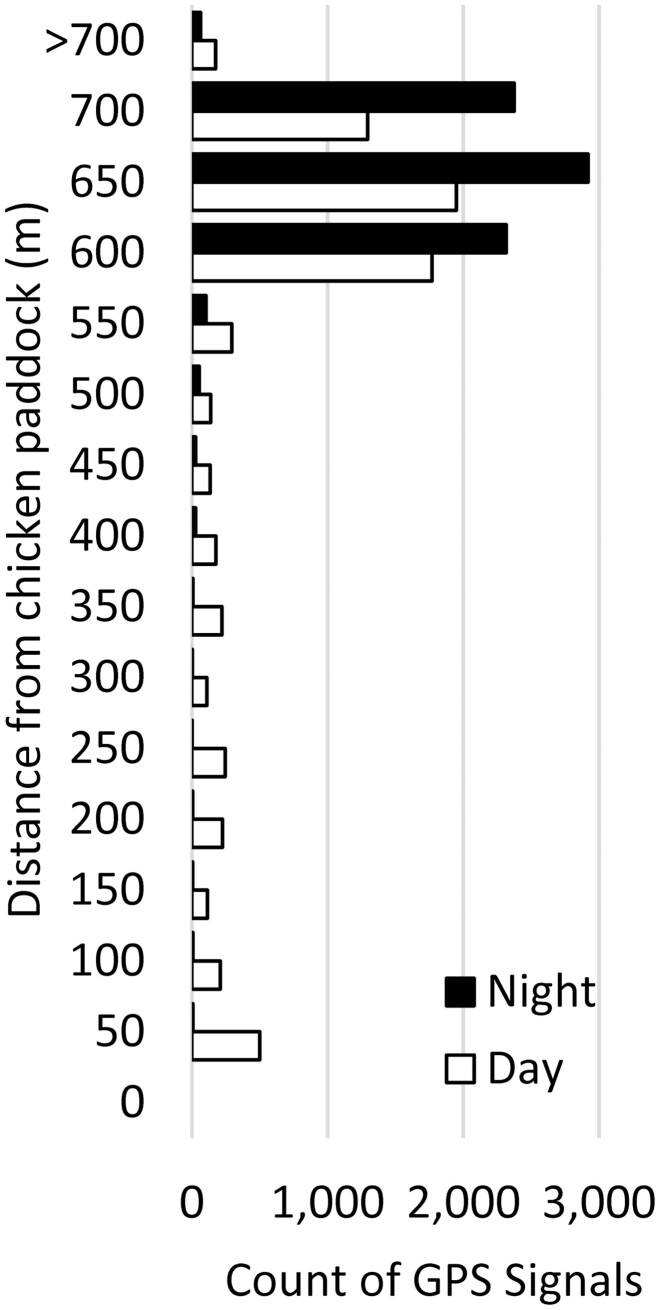
Distance from their chicken paddock for each location generated by LGD GPS collars. The data were grouped into 15 bins of 50 m each (0–700 m); 239 data points >700 m (ranging up to 1.55 km from the chicken paddock) were included in a single bin.

When allowed to roam across the whole property, the two LGDs spent the majority of their time together around the farmhouse, which was 550–700 m (Figure 3) from the position of their chicken paddock as it shifted over time (Paddocks M1–4; Figure 1). Most GPS locations were within 75 m of the farmhouse (82% of all locations, night: 96% and day: 66%), while only a minority of locations were within 75 m of their chicken paddock (3.3% of all locations, night: 0.1% and day: 6.6%). For both LGDs, there was a significant difference between day and night in the distance from their chicken paddock (Coco Kruskal–Wallis $\chi_{1}^{2}$ = 117.52, *P* < 0.001; Hiro Kruskal–Wallis $\chi_{1}^{2}$ = 10.38, *P* = 0.001), reflecting more time spent around the chickens during the daytime when the farm manager and two pet dogs moved about the paddocks collecting eggs. Both dogs traveled slower (Coco: Kruskal–Wallis $\chi_{1}^{2}$ = 12.93, *P* < 0.001, Hiro: Kruskal–Wallis $\chi_{1}^{2}$ = 99.0, *P* < 0.001) and remained closer to the farmhouse (Coco: Kruskal–Wallis $\chi_{1}^{2}$ = 284.2, *P* < 0.001, Hiro: Kruskal–Wallis $\chi_{1}^{2}$ = 6.73, *P* = 0.009) for nights with spotlights (switched on) than for nights without the spotlights (switched off).

### Does the Presence of LGDs Increase Paddock Use by Chickens?

We recorded a total of 15,373 sightings of chickens on the downward-facing cameras placed out in the Maremma paddocks, with only 0.27% of these being outside daylight hours (immediately before dawn). There was a strong influence of shade availability on chicken activity across the paddock (*t* = 8.05, *P* < 0.001; greater activity in more shaded parts of the paddock), and an effect of distance from the caravan (*t* = –3.31, *P* = 0.001; greater activity around the laying caravan). When these differences were accounted for by simultaneous inclusion in the analyses, there was no significant influence of LGD presence on chicken activity (*z* = –0.14, *P* = 0.999), and there was no significant interaction between LGD presence and distance from the caravan (*t* = –0.25, *P* = 0.803).

### Is Fox Activity Influenced by the Presence of Free-Roaming LGDs or Motion-Activated Sensor Spotlights?

No fox predation events occurred during the period of our study. A total of 40 fox sightings were recorded over the 46-d monitoring period, including at the perimeter of all three paddocks and at least once on each of the four spotlight camera traps. It was not possible to identify the numbers of individual foxes from the photographs as they generally lack unique identifying features, although at least two individuals were seen (one with a distinctive damaged tail). There was no significant difference in the incidence of fox sightings across the four treatment combinations (“dogs in”/”dogs out” combined with “spotlights on”/”spotlights off”, $\chi_{3}^{2}$ = 6.07, *P* = 0.108; Table 1) or for pairwise comparison of LGD treatment alone (for nights with spotlights off: $\chi_{1}^{2}$ = 1.56, *P* = 0.211). However, there were significantly fewer fox sightings for nights with dogs out + spotlights compared with spotlights alone ($\chi_{1}^{2}$ = 3.90, *P* = 0.048): only two fox sightings were recorded over the 8 nights of monitoring with dogs out + spotlights compared with 13 fox sightings over 13 nights with spotlights alone (Table 1).

**Table 1.** Summary of red fox (*Vulpes vulpes*) sightings across 16 camera traps positioned around the chicken paddocks (*n* = 12 camera traps) or adjacent to motion-activated spotlight positions (*n* = 4 camera traps) on nights with combinations of Maremma LGD treatment (confined to their chicken paddock “dogs in” or free-roaming the property “dogs out”) and motion-activated spotlight treatment (“spotlights on”/“spotlights off”). Chi-squared analyses were carried out for LGD treatment nights (“dogs in” vs. “dogs out” on nights without spotlights), or spotlight treatment nights (“spotlights on” vs. “spotlights off” for nights with the dogs out).

Treatment	Dogs	Spotlights	Duration of monitoring (nights)	Number of fox sightings	Trap rate	*χ* ^2^	*P*
Nil	In	Off	16	13	0.81	} 1.56	0.211
Dogs	Out	Off	9	12	1.33		
Spotlights	In	On	13	13	1.00	} 3.90	0.048
Dogs + spotlights	Out	On	8	2	0.25		
Total			46	40		6.07	0.108

On another 19 occasions, the 1 min time-lapse photos indicated that the spotlight had been activated but only twice a fox was seen on the camera trap within 1 minute of this activation. The only other nocturnal species recorded was the western gray kangaroo, which was infrequently sighted (only 4 events at night), suggesting a strong likelihood that these 19 triggers also indicated fox activity that was not detected by the camera traps. On one occasion a LGD was sighted on the same camera trap 15 min after a fox sighting.

### What Factors Influence the Effectiveness of LGDs? A Poultry Farmer Survey

There were 59 respondents to the survey, with approximately equal numbers of respondents from Australia/New Zealand (*n* = 27) and North America (USA and Canada, *n* = 26); 6 respondents did not give sufficient information to identify their location. Respondents identified that they currently had an average of 2.39 ± 2.42 (range 0–15) LGDs, and an average of 743 ± 1,596 (range 10–7,000) head of poultry. There was no difference in poultry numbers between Australian and North American respondents (two-sample *t*-test assuming unequal variances *t*_19.35_ = 1.48, *P* = 0.155). Respondents had owned their LGD/s to an average of 7.9 ± 9.0 yr (*n* = 54 respondents, range 1–35 yr). Most (56) respondents were using LGDs at the time of the survey, one formerly used them but was not at the time of the survey, and two had never used them. Nine breeds were reported (Table 2a), with Maremmas making up 56% of all LGDs currently being used (*n* = 28 respondents, 83% of dogs reported by respondents from Australia). The most commonly reported poultry predators (Table 2b) were foxes (identified by 66% of all respondents, including 85% of Australian respondents), while coyotes were reported by 77% of North American respondents. Raptors were also commonly reported as poultry predators (64% of all respondents).

**Table 2.** (a) LGD breeds (numbers of dogs) and (b) types of poultry predators reported by 59 respondents to the LGDs survey, grouped by reported region (one New Zealand respondent grouped with Australia).

	Australia	N America	Not disclosed	Total
a. Livestock Guardian Dog breeds	Numbers of dogs			
Anatolian Shepherd	6	4	1	11
Armenian Gampr		7		8
Akbash		1		1
Central Asian Shepherd		7		7
German Shepherd	1			1
Great Pyrenees		8		8
Maremma	62	5	7	74
One Karakachan and one Anatolian		2		2
Sarplaninac		9		9
Unknown or mixed breed	5	6		11
Total number of dogs	75	49	8	132
b. Main predators	Numbers of respondents			
Birds (e.g., *Aquila* spp., *Buteo* spp., *Corvus* spp.)	16	18	4	38
Canids				
Domestic dogs (*Canis familiaris*)	2	8		10
Wild dogs/dingoes (*Canis familiaris*)	4	–		4
Coyotes (*Canis latrans*)	–	20		20
Fox (*Vulpes vulpes* or unidentified species)	23	10	6	39
Felids (e.g., *Felis catus, Lynx rufus*, *Puma concolor*)	4	10	1	15
Bears (e.g., *Ursus americanus*, *U. arctos*)	–	7		7
Raccoon (*Procyon lotor*)	–	12		12
Opossum (*Didelphis virginiana*)	–	3		3
Skunks (family Mephitidae)	–	6		6
Quoll (*Dasyurus* spp.)	1	–		1
Reptiles (e.g., snakes, goannas *Varanus* spp.)	3	1		4
Humans	1	2		3
Total number of respondents	27	26	6	59

– indicates predator species not found in that region.

Although most of the large-scale commercial farms did not provide numbers, some respondents indicated actual numbers for losses they had incurred due to predators:

*16 losses before the dog, 2 after* (small scale breeder with an undisclosed number of a variety of poultry)

*Used to lose about 10 % before the dog* (with 33 chickens)

*No losses since new dog arrived. Previous dog passed away we were without a LGD for over a month. We lost 8 chickens during that time.* (with 25 chickens)

**Figure 4.**
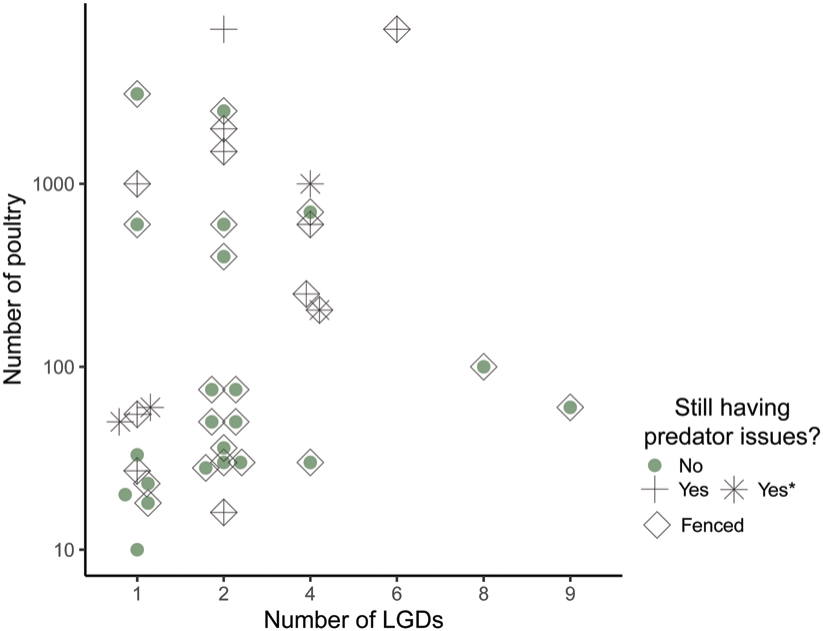
Scatterplot of data for 54 survey respondents reporting whether they had persisting predator issues (yes or no), shown by the number of LGDs, number of poultry, and whether the poultry area was fenced. * Four respondents reported persisting predator issues for aerial predators only.

When asked “Do you currently have any issues with predators?”, about half (46%) of the 54 respondents who answered this question selected “yes” (four of these indicated that their issues were only with aerial predators), while the remainder (54%) selected “no”. All 54 respondents currently had LGDs. None of the respondents who reported persisting predator issues identified changes in chicken behavior, productivity, damage to fences, or injuries to pets or working animals; instead, their descriptions were directly related to issues of poultry predation. The strongest factor correlated with reports of persisting predator issues was the number of poultry respondents had (*z* = 2.13, *P* = 0.034), with farmers that owned ≥100 birds more likely to report issues. There was no influence of the number of LGD (*z* = 0.04, *P* = 0.964) or whether the poultry were kept in a fenced area (*z* = 1.40, *P* = 0.162) on reports of persisting predator issues (Figure 4).

When asked “Would you say the dogs are more bonded to …” around half (47%) of respondents selected “livestock” while the other half (53%) selected “people”; there was no significant difference between these two groups in reports of persisting predator issues ($\chi_{1}^{2}$= 0.07, *P* =0.790; 49 respondents). There was also no difference in reports of persisting predator issues between farms where the LGDs received <1 h per week, <1 h per day, or >1 h per day of human interaction ($\chi_{2}^{2}$ = 0.17, *P* = 0.683; 51 respondents), by dog breed (Maremma or other) ($\chi_{1}^{2}$ = 0.06, *P* = 0.805; 53 respondents), nor whether the dogs worked alone or in groups ($\chi_{1}^{2}$ = 0.16, *P* = 0.691; 51 respondents). When asked what advice they would give to farmers using LGDs for the first time, those who mentioned LGD training were less likely to report persisting predator issues ($\chi_{1}^{2}$ = 5.85, *P* = 0.016; 49 respondents).

Three quarters (72%; 36 of 60) of the respondents indicated that they believed LGDs did not seem to affect the movements of the chickens and where they spend their time in the paddock, with the remaining 28% selecting the option that the poultry used more of the space available to them. Most (84%; 42 of 50) respondents believed the LGDs would still display protective behavior towards poultry if they were no longer confined to a fenced area with them. Compared with LGDs that did not spend all their time with the livestock, LGDs that spent all their time with livestock were more likely to have injured or killed livestock ($\chi_{1}^{2}$ = 7.15, *P* = 0.007; 51 respondents).

Most (49/51) respondents answered that they believed LGDs were effective in protecting poultry; the two respondents who did not give a clear endorsement wrote:

Ground predators yes. Aerial predators no.

*No, the predators (bear, coyotes, hawks) still come around, but they don’t stay long. [the dog] acts more as an early warning system to us so we can gather the chickens into the covered runs (hawks), or scare off the predators who either ignore her (bears) or act uncertain, hanging around in the underbrush (a couple of juvenile coyotes when she was about a year old). The one predator that we had had problems with pacing the fenceline (bobcat) now stays far away— we have seen traces (scat) … over 100ft from the fenceline.*

Most (49/50) respondents answered “yes” that they would recommend the use of LGDs to other chicken farmers.

*They are extremely effective in our situation/ environment. I’m not sure mobile poultry systems can be sustainable & promote good welfare outcomes without them.*

*I find that other than stopping and deterring predator attacks they also help keep order in the paddocks as in they sort out the squabbles and fights between animals.*

*Can’t farm pastured chickens without them.*

*I sleep better knowing that my birds are protected, I don’t have to listen for every chicken noise. This is worth a lot!*

*No other way to protect from aerial predators, don’t have to move electric netting all the time, nicer for hens to be truly free range.*

*They are the only thing that works.*

These responses highlight the generally positive and enthusiastic opinions given by the survey respondents on the use of LGDs in their industry. The only negative opinion received was that LGDs were too expensive in the producer’s local area:

The dogs here are typically very expensive (> $1000/yr between food and medical bills). I am a small chicken farmer, so can’t justify that as an expense. The electric fence was probably the better investment. Once she is totally trustworthy with free-ranging chickens, I may change my mind, and certainly for a large operation it may be worth it, but between her showing some interest in chasing a flappy hen (she’s always been on leash and has been redirected) and the chickens instinctively being afraid of her, I’d say it’s not a “plug-and-play” solution. It definitely takes training of the dog, and habituation of each new chicken to the dog(s), to get them (all) to be calm around each other.

A second respondent indicated that they would only recommend the dogs for properties >10 acres and with few neighbors, on account of the dog’s barking and likely noise complaints.

## DISCUSSION

We worked with a commercial egg producer to understand the role of guardian animals in protecting free-ranging pastured hens. The LGDs were let out of the small chicken enclosures for 2–3 d a week and showed a preference for being at the farm residence, but this had no effect on the behavior of the chickens and there was no additional fox activity around the chicken paddocks as a consequence. Adding motion-activated spotlights supplemented the LGDs when they were free-roaming across the property, with fewer foxes recorded. We supported these case study data with a survey of poultry farmers who have experience with LGDs via an online survey to identify factors that influenced the dogs’ effectiveness (i.e., reduction in poultry predation). Although the property we studied only had two LGDs, this is representative of the use of LGDs for guarding poultry (average of 2.39 LGDs per property reported by other poultry owners surveyed). Individual differences in LGD behavior (Roddick et al., 2022) and the unique situations found on each property (Campbell et al., 2021) somewhat limit generalization of this study, although some underlying findings can be informative about the behavior of LGDs around poultry.

### Are LGDs Bonded to Their Chickens When They are Free to Roam?

Dogs are social animals, and appropriate social bonding is paramount to ensuring LGDs will be suited, and can be trusted, to protect livestock (van Bommel, 2010). Proper socialization with livestock as a puppy will increase the likelihood of it voluntarily remaining with livestock (Coppinger and Coppinger, 2001; Dawydiak and Sims, 2004; van Bommel and Johnson, 2014). Although an LGD bonded too strongly with humans may have a weaker bond with livestock, allowing humans sufficiently close to catch and handle it is necessary, for example, for veterinary treatment (van Bommel, 2010). A balance between bonding with livestock and people is therefore essential to ensure that they are effective in their role. For our survey of poultry farmers, there was no relationship between their perceptions of LGD level of socialization with people (compared with the livestock) and whether they reported persisting predator issues. There was also no relationship between levels of human contact received by LGDs and persisting predator issues. While bonding with people does not preclude LGDs being protective of livestock, bonding with people could compromise their guarding if it influences how far they wander from livestock (Landry et al., 2020), especially where livestock are kept at a distance from where people reside.

We found no indication that the two Maremmas that were the focus of the present study were bonded to their chickens, and when they are free to roam the property, they spent minimal time around the chicken paddocks. When released, the LGDs spent 82.5% of their time within 75 m of the farmhouse, and only 3.3% of their time within 75 m of their chicken paddock, and even less time (0.09%) near the paddock during the night, at the time when foxes were active on the property. This result contradicts the perceptions of poultry farmers, with 80% of survey respondents believing that their dogs would display protective behavior toward poultry, even when the dogs were no longer confined to their paddock. This result suggests a possible disconnect between producer beliefs and expectations towards their LGDs versus the realities of potential distractions and bonding imbalances. Bonding with humans is not desirable because some studies suggest that LGDs do not establish territorial boundaries from which predators are excluded (Allen et al., 2017; Roddick et al., 2022; but see van Bommel and Johnson, 2015) and therefore the direct presence of the LGD itself is important for protecting livestock. The reality is that LGDs require training (Rust et al., 2013; survey respondents in the present study) and some are likely to need to be restrained to remain with the livestock they are protecting (van Bommel, 2010), although the small size of many poultry production facilities raises the issue of balancing the welfare of the LGDs themselves (providing exercise for mobility and stimulation) as well as that of the livestock.

### Does the Presence of LGDs Increase Paddock Use by Chickens?

Chickens did not alter their paddock space use with LGD presence, with no increase in overall chicken activity, and no interaction between LGD presence and distance from the safety of the laying caravan (when accounting for shade availability). This result supports the observations of most survey respondents, 72% of whom believed LGDs did not seem to affect poultry movements or where they spend their time. In our study, chickens were only active in the paddock during daylight hours, when raptors (known predators of chickens, Conroy et al., 2005) were also active. Published research suggests that chickens may be able to anticipate future events, perceive time intervals, and are more cognitively, emotionally, and behaviorally complex than was once thought (Marino, 2017; Garnham and Lovlie, 2018). The lack of effect of LGD presence on chicken paddock space use was therefore somewhat surprising; it could indicate that raptor wariness did not shape the chickens’ behavior, the dogs were not gone long enough for the chickens to adjust their activity patterns, or that the chickens did not perceive reduced predator risk in the presence of the dogs.

### Is Fox Activity Influenced by the Presence of Free-Roaming LGDs or Motion-Activated Sensor Spotlights?

Free-roaming LGDs and motion-activated spotlights were the strongest combination for fox deterrence. Although they spent most of their time around the farmhouse, 4% of location records showed that the dogs patrolled the whole property. There were fewer fox records for the dogs + spotlights treatment suggesting that foxes avoided the property on these nights, which might explain why the dogs showed slower average speeds and were positioned closer to the farmhouse on nights with the motion-activated spotlights switched on. Foxes will readily habituate to lighting deterrents (Khorozyan and Waltert, 2019), including motion-activated spotlights such as the ones used in the present study (Hall and Fleming, 2021). The spotlights might alert LGDs to the presence of foxes, or the spotlights might reinforce the deterrence value of the dogs’ presence, with the combination of dogs + spotlights having the least likelihood of foxes habituating to the spotlights on their own.

### What Factors Influence the Effectiveness of LGDs? A Poultry Farmer Survey

Most (49/51) survey respondents considered that LGDs were effective in protecting poultry, as is expected for voluntary participation in a survey on a topic that people strongly believe in. This aligns with previous surveys in other countries that have consistently shown high levels of perceived effectiveness reported by producers (Green et al., 1984; Green and Woodruff, 1989; Marker et al., 2005; Otstavel et al., 2009; Potgieter et al., 2013; Rust et al., 2013). Analysis of a record of losses before and after acquiring LGDs suggests that effectiveness rates for LGDs remain high over time (Coppinger et al., 1988; Andelt and Hopper, 2000; van Bommel and Johnson, 2012). In the only large-scale Australian producer survey that has been conducted, 68% of producers who obtained LGDs as a result of predation issues reported that predation had ceased completely once the dogs were introduced, while a further 30% reported a reduction in predation (van Bommel and Johnson, 2012). Only 6% of respondents in the current study were able to provide before and after predation rates and the more useful response was judged to be whether the respondents reported persisting predator issues.

The number of poultry under their care is likely to influence the efficacy of LGDs, with producers managing ≥100 birds more likely to report persisting predator issues. While we found no influence of the number of LGD, previous research indicated that the number of LGDs, as well as the number of livestock under their care, have the closest correlation to predict reduction in predation (van Bommel and Johnson, 2012). Larger numbers of livestock are likely to require more LGDs to adequately protect them if they are spread over a greater area, or would require a combination of methods to increase their effectiveness. We found no significant effect of whether poultry was fenced on reports of persisting predator issues, although we did not specify the type of fencing or asked if it was likely to be predator proof. Reviews indicate that electrified fencing has the strongest and most persisting effect as a predator deterrent (Miller et al., 2016; Moreira-Arce et al., 2018; Khorozyan and Waltert, 2019), while the efficacy of LGDs, which has the largest overall effect as a predator deterrent (Van Eeden et al., 2018a), sometimes shows ambiguous results (Miller et al., 2016; Khorozyan and Waltert, 2019). The combination of LGDs with fencing to reduce the area over which birds move is likely to be the most successful combination. For example, irrespective of the number of dogs employed, the efficiency of LGDs against wolf (*Canis lupus*) attacks was greatest when sheep were also confined at night (Espuno et al., 2004). Similarly, a combination of electric fencing and guardian animals was used effectively on our study property, and most (70%) of the poultry farmers responding to our survey similarly indicated that they also used fencing for managing their flocks.

### Further Research

Results of this pilot study around human bonding and behavior toward their livestock warrant further testing with other LGDs. Although they had been brought up with poultry and were expected to have shown strong bonds to their livestock, our data were generated from only two sibling dogs who were scarcely found separated. We have also been limited to working with experimental questions consistent with commercial farm husbandry and layout, but it would be worth expanding on this work, where logistics permits. While there has been very limited published data showing the behavior of guardian animals around poultry, the substantial (and rapid) engagement with our survey suggests that there is a reasonable number of farmers currently using LGDs for the protection of their poultry and the relationships between dog and poultry are worth further investigation. For example, developing some standardized measures of the degree of human and livestock bonding in LGDs would be a valuable step in comparing individual personality differences for the dogs as well as comparing their behavior for different types of livestock.

## CONCLUSION

While the ratio of LGD to livestock continues to be the best metric of success in predation reduction (e.g., Landry et al., 2020), LGD bonding and individual behavioral responses will also contribute to their successful use in agriculture. Continuous close bonding and socialization with humans are likely to result in long periods of LGD absence from their livestock. Given that some studies report that it is the direct presence of the dogs that appears to confer livestock protection, their absence could therefore expose livestock to greater predation risk. However, there is also a substantial animal welfare consideration for keeping LGDs in small pens with chickens. In this pilot study, despite high socialization and bonding with humans, the combination of electric fencing and guardian animals proved effective, with no evidence of chicken predation by foxes during the study period or over the previous year. The effectiveness of the LGDs was further supported by the introduction of motion-activated spotlights, which would serve to disrupt predator activities and reinforce wariness towards the dogs’ presence.

## Supplementary Material

txad014_suppl_Supplementary_Material
