# Supplementary material for: Chicken’s best friend? Livestock guardian dog bonding with free-ranging chickens
Source: Transl Anim Sci. 2023 Feb 7;7(1):txad014. doi: 10.1093/tas/txad014 (PMC9977226; doi:10.1093/tas/txad014)
Supplement: txad014_suppl_Supplementary_Material [file txad014_suppl_supplementary_material.pdf]

SUPPLEMENTARY MATERIAL

Appendix A

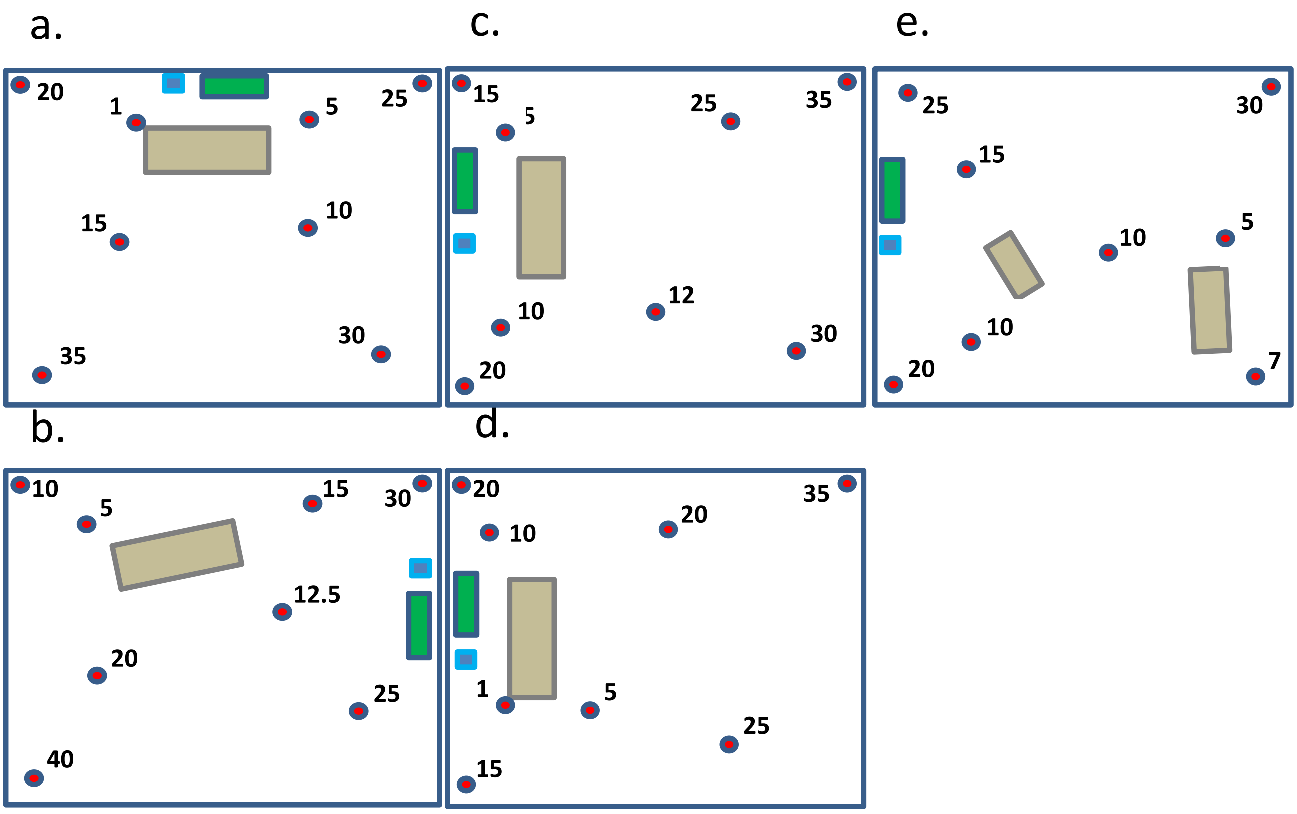

Figure S1. Layout of camera traps in the chicken paddocks. Paddock 'M' contained two Maremma Livestock Guardian Dogs and 450 ~9-month-old chickens; camera positions for (**a**) M1, 9–23 July or (**b**) M2, 23–29 July. Paddock 'A' contained two alpacas and 500 ~6-month-old chickens; camera positions for (**c**) A1, 9–16 July or (**d**) A2, 16–29 July. Paddock 'N' contained no guardian animal and the oldest chickens at ~18-months-old. Paddock N was not moved during the study as the chickens were being sold off; there were 68 chickens for the first 12 days of monitoring, 47 for the next day, and then 25 chickens for the last 2 days of monitoring for this paddock. (**e**) Camera positions for Paddock N 9–24 July. For each diagram, the blue boundary line indicates the electric fence. Red dots indicate camera trap placement. Grey rectangle indicates the chicken caravan. Green rectangle indicates the chicken feeding troughs. Blue square indicates the water trough. Numbers next to each red dot indicate distance (m) from the nearest chicken caravan corner.

The summed number of chickens seen on each camera each hour ('chicken activity') was calculated as a proportion of the total number of chickens in each paddock (to account for differences between the three paddocks and reduction in numbers over time for Paddock N). Using a generalized linear mixed-model analysis (*glm* function in 'lme4' package in R), chicken activity (dependent value) was compared by paddock treatment (Paddocks A, N, and M with and without the Maremmas). The proportion of photos showing >50% field of view with shade present was calculated for each hour for each camera, and the distance between the camera and the caravan (m), were included as covariates. We used a Tukey pair-wise comparison in the R package 'emmeans' (Lenth et al., 2018) to estimate pairwise differences in the *treatment x distance from*

caravan interaction terms, and used *ggpredict* in the R package ‘ggeffects’ (Lüdecke et al., 2020) to predict the relationships, standardizing for the differences in shade availability (held at an average value).

Across all treatments, there was greater activity around the caravan and less activity further away (t = –5.35, p<0.001). In terms of the interaction between livestock guardian animal treatment and distance from the caravan, there were significant differences between Paddock N and the other three treatments (p<0.001 for each pairwise comparison), with chickens in paddock N unlikely to be observed more than 15 m from the caravan. Chickens in Paddock A were more likely to be observed further away from the caravan than chickens in Paddock M (LGDs present z = –2.94, p=0.017, LGDs absent z = –2.80, p=0.026), but there was no significant difference for Paddock M with LGD presence/absence (z = –0.14, p=0.999).

Unfortunately, this experimental design did not allow us to identify differences that could be attributed to the differences in presence or type of livestock guardian animals. Our ‘control’ paddock (no guardian animal) did not have the same conditions as the two paddocks with guardian animals, and we could not distinguish an effect of the guardian animals from age of the chickens, paddock novelty (both Paddock M and Paddock A were moved every 2–3 weeks to an adjacent 50 x 50 m area), or chicken number and therefore density of the chickens.

Lenth, R., H. Singmann, J. Love, P. Buerkner, and M. Herve. 2018. Emmeans: Estimated marginal means, aka least-squares means. R package version 1(1):3.

Lüdecke, D., F. Aust, S. Crawley, and M. Ben-Shachar. 2020. Package ‘ggeffects’, CRAN.

Appendix B

Survey Questions

Livestock Guardian Dogs – How they protect Livestock and the effects on fox movements

1. General

Do you consent to us recording your address? (so that we can compare your data with other farms in the same shire and compare your responses with published information about the presence of predators in these locations) ☐ yes, ☐ no

- Address: ____
- What size is your property? ____ hectares
- What sort of topography and vegetation is your property?

  ☐ Flat, ☐ Hilly, ☐ marked topography (lots of refuges for predators)

  Vegetation within 0.5km of the pens/paddocks:

  ☐ Completely cleared, ☐ <20%, ☐ 20-50%, ☐ >50% vegetation cover

- Which breed of chicken do you have ____

  and how many ____ ?

- How long has the farm been in operation? ____________________________________ years
- What are the main livestock predators that you deal with? __________________________

  ____

  ____

- Have you ever used livestock guardian dogs on your property?

  ☐ I currently use them on my property, ☐ I used to use them on my property but do not currently, ☐ I have never used them

  If 1, move to section 2

  If 2, move to section 3

  If 3, move to section 4

2. Those with current LGDs

Origins:

- How did you first become aware of the use of livestock guardian dogs?

  ☐ word of mouth, ☐ newspaper or other media, ☐ other

  ____

  ____

- Why did you decide to use them on your farm?

  ____

  ____

- Where did you get the dogs from?

  ☐ direct from a breeder, ☐ newspaper or other media, ☐ other

  ____

  ____

- How many livestock guardian dogs do you currently have? ________________________

  What breed of livestock guardian dog do you have?

  ☐ Maremma, ☐ other

  ____

- For how many years have you had them in total? ___________________________ years

- How many livestock guardian dogs have you had in total? ________________________

  ____

  ____

Predators:

- Do you currently have any issues with predators?

____

____

- What methods of predator control do you currently use apart from LGDs?

____

____

- Are you happy with the results of these methods?

____

____

Dogs at Work:

- What is the setup size and layout of the area that the dogs are working in?

____

____

- Is the area

☐ fenced, ☐ open

- If fenced, can the dogs move through the fences?

☐ yes, ☐ no

- Do the dogs work alone, or in groups?

☐ alone, ☐ in groups

- Do the dogs spend all of their time with their livestock?

☐ yes, ☐ no

- How much human interaction do the dogs receive?

☐ less than 1 hour per week, ☐ less than 1 hour per day, ☐ more than 1 hour per day

- Would you say the dogs are more bonded to

☐ people ☐ the livestock?

- If the dogs were not confined to their paddock do you believe they would still display protective behaviour towards the chickens?

☐ yes, ☐ no

- How do the chickens interact/act around the dogs?

☐ they don't seem to notice the dogs, ☐ they appear to use more of their enclosure, ☐ other

____

____

- Do you think the dogs have an effect on the movements of the chickens and where they spend their time in the paddock?

☐ they don't seem to notice the dogs, ☐ Yes they use more of their enclosure

Problems:

- Have any of your dogs ever killed or injured any livestock?

☐ yes, ☐ no

- Have any of your dogs exhibited aggression towards humans?

☐ yes, ☐ no

- Have any of your dogs exhibited any other behavioural problems?

____

____

Effectiveness:

- Did you record the numbers of chickens lost before and after introducing the livestock guardian dogs?

____

____

- Do you believe the dogs to be effective in their role? Why?

____

____

- Do you believe that they keep predators away?

____

____

- Do you believe the dogs to be worth the price of purchase/training/upkeep?

☐ yes, ☐ no

- Would you recommend them to other chicken farmers? Why/why not?

☐ yes, ☐ no

____

____

- What advice would you give to farmers bringing in LGDs for the first time?

____

____

____

____

Your survey is now completed. Thank you for taking part.

3. Those without current LGDs who did have them

Origins:

- How did you first become aware of the use of livestock guardian dogs?

  ☐ word of mouth, ☐ newspaper or other media, ☐ other

  ____

  ____

- Why did you decide to use them on your farm?

  ____

  ____

- Where did you get the dogs from?

  ☐ direct from a breeder, ☐ newspaper or other media, ☐ other

  ____

  ____

- How many livestock guardian dogs did you have? ______________________________

  What breed of livestock guardian dog did you have?

  ☐ Maremma, ☐ other

  ____

- For how many years did you had them in total? ____________________________ years

- How many livestock guardian dogs have you had in total? ________________________

  ____

  ____

Predators:

- Do you currently have any issues with predators?

____

____

- What methods of predator control do you currently use?

____

____

- Are you happy with the results of these methods?

____

____

- Did you use this method/s when you used LGDs?

☐ yes, ☐ no

Dogs at Work:

- What is the setup size and layout of the area that the dogs worked in?

____

____

- Was the area

☐ fenced, ☐ open

- If fenced, could the dogs move through the fences?

☐ yes, ☐ no

- Did the dogs work alone, or in groups?

☐ alone, ☐ in groups

- Did the dogs spend all of their time with their livestock?

☐ yes, ☐ no

- How much human interaction did the dogs receive?

  ☐ less than 1 hour per week, ☐ less than 1 hour per day, ☐ more than 1 hour per day

- Would you say the dogs were more bonded to

  ☐ people ☐ the livestock?

- If the dogs were not confined to their paddock do you believe they would still have displayed protective behaviour towards the chickens?

  ☐ yes, ☐ no

- How did the chickens interact/act around the dogs?

  ☐ they don't seem to notice the dogs, ☐ they appear to use more of their enclosure, ☐ other

____

____

- Do you think the dogs had an effect on the movements of the chickens and where they spent their time in the paddock?

  ☐ they didn't seem to notice the dogs, ☐ Yes they used more of their enclosure

Problems:

- Did any of your dogs ever kill or injure any livestock?

  ☐ yes, ☐ no

- Did any of your dogs exhibit aggression towards humans?

  ☐ yes, ☐ no

- Did any of your dogs exhibit any other behavioural problems?

____

____

Effectiveness:

- Did you record the numbers of chickens lost before and after introducing the livestock guardian dogs?

  ____

  ____

- Do you believe the dogs were effective in their role? Why?

  ____

  ____

- Do you believe that they kept predators away?

  ____

  ____

- Why do you no longer have them?

  ____

  ____

- Would you consider using them again? Why/why not?

  ☐ yes, ☐ no

  ____

  ____

- Do you believe the dogs to be worth the price of purchase/training/upkeep?

  ☐ yes, ☐ no

- Would you recommend them to other chicken farmers? Why/why not?

☐ yes, ☐ no

____

____

- What advice would you give to farmers bringing in LGDs for the first time?

____

____

____

____

Your survey is now completed. Thank you for taking part.

4. Those who have never had LGDs

- Do you currently have any issues with predators?

____

____

- What methods of predator control do you currently use?

____

____

- Are you happy with the results of these methods?

____

____

- Are you aware of the use of livestock guardian dogs in farming?

☐ yes, ☐ no

- If so, where did you hear about it?

☐ word of mouth, ☐ newspaper or other media, ☐ other

____

____

- Have you considered using them to protect your chickens?

____

____

- What would be your biggest concern in deciding whether to try livestock guardian dogs?

☐ cost ☐ training/time ☐ effectiveness ☐ risk to livestock ☐ other

____

____

Your survey is now completed. Thank you for taking part.
